# Endomyocardial Biopsy of Right Atrial Angiosarcoma Guided by Intracardiac Echocardiography

**DOI:** 10.4061/2010/681726

**Published:** 2010-05-24

**Authors:** Suman S. Kuppahally, Sheldon E. Litwin, Andrew D. Michaels

**Affiliations:** Department of Medicine, Division of Cardiology, The University of Utah, Salt Lake City, UT 84132, USA

## Abstract

We report a case of a 22-year-old female who presented with pericardial effusion and cardiac tamponade. She was diagnosed with a right atrial mass by computed tomography and was referred to our institution for biopsy of this mass. Transcatheter biopsy was performed with intracardiac echocardiography (ICE) guidance, avoiding the need for transesophageal echocardiography or surgery to obtain the biopsy. ICE for transcatheter biopsy of an intracardiac mass is an attractive modality which provides precise localization of the cardiac structures.

## 1. Case Report

**Figure 1:**
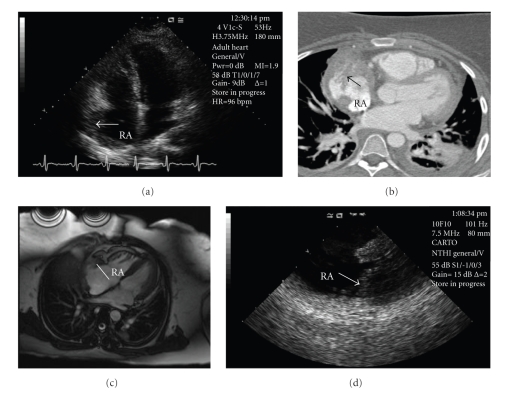
Right atrial mass imaging by different modalities: (a) Transthoracic echocardiography, apical four-chamber view. Thickening of the right atrial wall (arrow). (b) Chest CT, axial view. Enhancing mass (arrow) with papillary projections in the lateral wall of the right atrium. (c) Cardiac MRI, axial view. A heterogeneous, vascular mass involving the right atrial lateral wall. (d) Intracardiac echocardiography. Irregular mass (arrow) infiltrating the right atrial wall.

A 22-year-old female presented with dyspnea on exertion for 2 months and a syncopal episode. On presentation to an outside hospital, she was found to have a pericardial effusion with evidence of cardiac tamponade. An emergent pericardiocentesis was performed and 50 milliliters of bloody fluid were drained with immediate improvement in her hemodynamics. Cytology of pericardial fluid did not demonstrate malignancy. Transthoracic echocardiogram showed mild thickening of the right atrial wall (Figure 1(a)). Electrocardiographic-gated computed tomography (CT) showed a large mass with a broad-based attachment to the free wall of the right atrium (RA). The mass filled most of the RA appendage with an irregularly shaped border (Figure 1(b)). The right coronary artery was not involved. A 1.5 Tesla cardiac MRI (Avanto, Siemens Medical Solutions, Malvern, PA) with gadolinium contrast showed a similar appearance of the mass without evidence of fat or any delayed enhancement (Figure 1(c)). She was referred to our institution for biopsy of the mass. 

**Figure 2:**
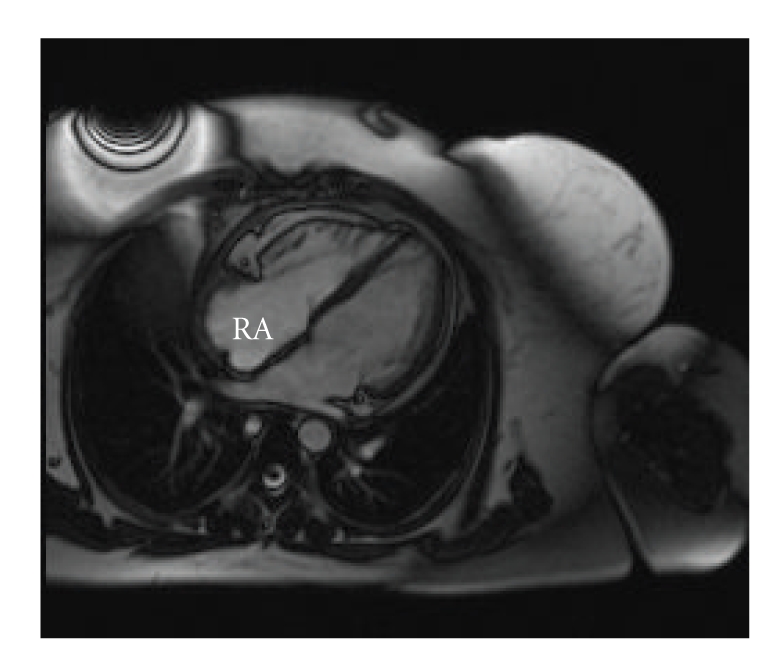
Cardiac MRI after chemotherapy of right atrial angiosarcoma showing significant resolution of the mass.

We performed percutaneous transcatheter biopsy of the RA mass using fluoroscopic and intracardiac echocardiographic (ICE) guidance (10F Sound-Star, Biosense Webster, Diamond Bar, CA). The tumor infiltrated the wall of RA free wall and was 1.5 centimeters in thickness. ICE showed papillary projections as seen on the CT scan (Figure 1(d)). Pathologic analysis showed a malignant angiosarcoma. She underwent 4 cycles of chemotherapy with doxorubicin and ifosfamide, and surveillance cardiac MRI showed significant resolution of the RA mass (Figure 2). 

## 2. Discussion

Cardiac sarcomas are rare primary malignant cardiac tumors. Of these, angiosarcomas typically arise in the right atrium as mural masses. Cardiac imaging by MRI has been useful in understanding the tissue characteristics of the tumor and in surveillance after therapy. The histological diagnosis of cardiac masses is made at autopsy or typically requires an open-chest procedure, often with cardiopulmonary bypass. Transcatheter biopsy of cardiac mass with transesophageal echocardiography guidance [1] requires conscious sedation or general anesthesia which is uncomfortable to the patient. The use of ICE for transcatheter biopsy of an intracardiac mass is an attractive modality, especially for right-sided structures [2]. It provides precise localization of the cardiac structures without significant artifacts and does not need general anesthesia. ICE may not be appropriate in patients with femoral venous or inferior vena caval thrombosis or in centers not experienced with ICE imaging.
